# Severe pulmonary hemorrhage in a 3‐week‐old neonate with COVID‐19 infection: A case report

**DOI:** 10.1002/ccr3.6189

**Published:** 2022-08-08

**Authors:** Anood Alassaf, Khaled Ellithy, Tejas Mehta, Walid Aljbawi, Hossamaldein Ali, Ashraf Soliman, Mohammed Al Amri, Abdulqadir J. Nashwan

**Affiliations:** ^1^ Pediatrics Department Sidra Medicine Doha Qatar; ^2^ Pediatrics Department Hamad Medical Corporation Doha Qatar; ^3^ Pediatric Intensive Care Unit Hamad Medical Corporation Doha Qatar; ^4^ Pharmacy Department Hamad Medical Corporation Doha Qatar; ^5^ Pediatric Endocrinology Department Hamad Medical Corporation Doha Qatar; ^6^ Pediatric Emergency Department Hamad Medical Corporation Doha Qatar; ^7^ Nursing Department Hamad Medical Corporation Doha Qatar

**Keywords:** COVID‐19, newborn, pulmonary hemorrhage, SARS‐CoV‐2, sepsis

## Abstract

Our patient is a 3‐week‐old female neonate, presented with complaints of low‐grade fever and a congested nose for one day. Eventually, she developed progressive desaturation, hypotension, and poor perfusion due to severe pulmonary hemorrhage. Then, she developed cardiac arrest and was declared dead.

## Graphical abstract

Pulmonary hemorrhage has been reported in adults but rarely in children. To the best of our knowledge, this is the youngest age at which a patient with COVID‐19 infection developed pulmonary hemorrhage with no other underlying cause of it.

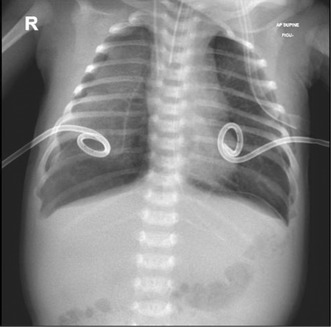

## 1 INTRODUCTION

During the SARS‐CoV‐2 pandemic, the majority of pediatric cases presented with lung involvement as the main disease, with the severity of symptoms ranging from mild pneumonia to severe lung injury and ARDS. Emerging studies found that some patients may experience uncommon complications, such as thrombotic or hemorrhagic episodes.^1^ The cases of pulmonary hemorrhage have been reported in adults with COVID‐19 infection; however, reports about similar presentations in pediatrics are rare. We present a case of a 3‐week‐old neonate with COVID‐19 infection and no other underlying comorbidities but a fatal pulmonary hemorrhage. Our case report demonstrates the unusual presentation of COVID‐19 infection in neonates and presents the challenges associated with it.

## 2 CASE PRESENTATION

**FIGURE 1:**
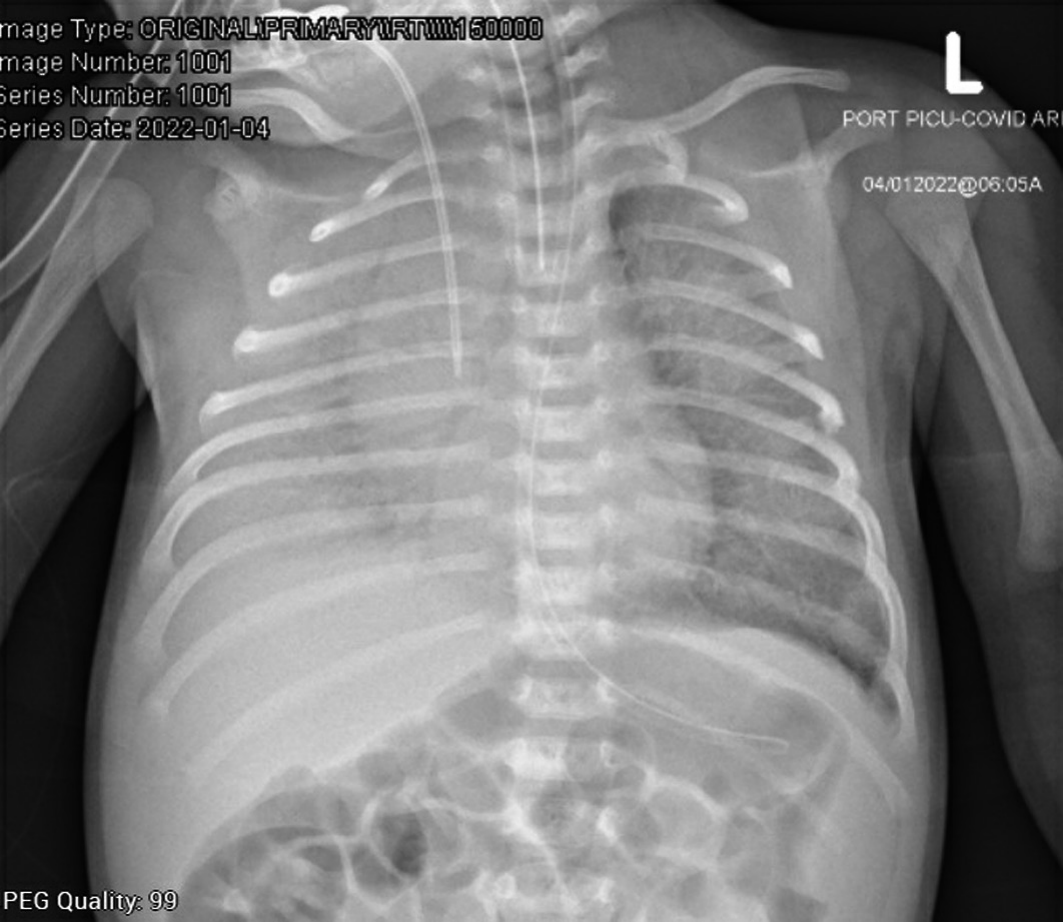
There is a diffuse bilateral alveolar infiltrate obscuring the right hemithorax. Central venous catheter, endotracheal tube, and nasogastric tubes are shown in the X‐ray.

**FIGURE 2:**
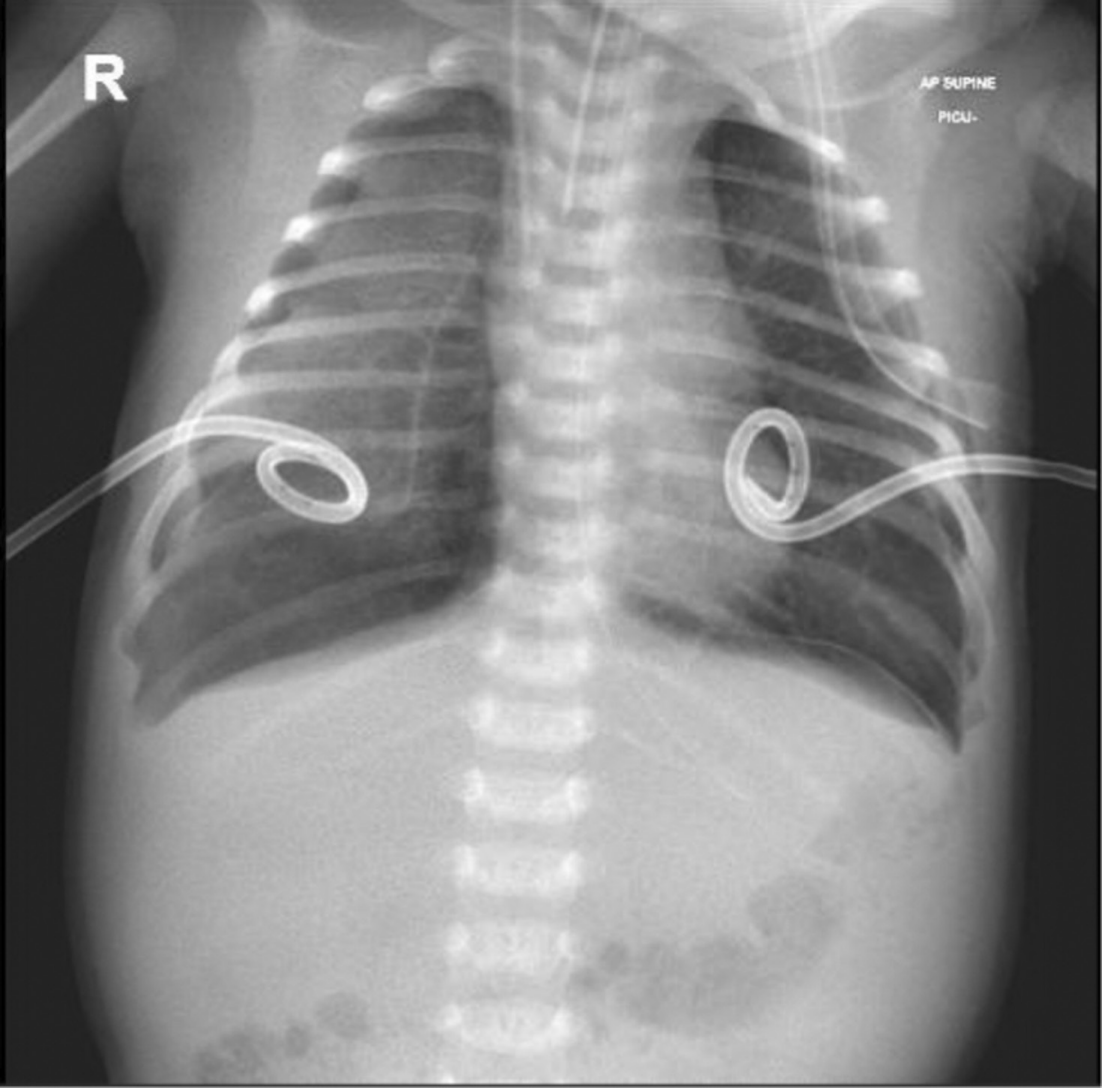
Bilateral partial pneumothoraxes, for which pigtail catheters were inserted and seen in place.

The patient is a 3‐week‐old female infant, a product of full‐term pregnancy and uneventful normal vaginal delivery. She was delivered to a 37‐year‐old healthy GBS‐negative mother. Her birth weight was appropriate for her gestational age, she did not require any resuscitation or oxygen therapy after birth, and her bilirubin before discharge from NICU was within normal range. Therefore, she was discharged with her mother after 24 h in a good condition. She presented to the emergency department with a high‐grade fever, hypoactivity, and poor oral intake for a one‐day duration. Physical examination at that time was significant for fever reaching 38.9°C and tachycardia, which improved after a bolus of 10 ml/kg normal saline 0.9%. The rest of the examination was unremarkable, the fever responded to antipyretics, and the patient was doing well. A full septic workup was done, and cultures from blood, urine, and CSF were taken. Her initial blood workup, including blood gas and CSF study, was reassuring (Table 1). Her SARS‐CoV‐2 RT‐PCR was positive (CT value 17.77). Both parents were COVID‐19 positive, and the father was symptomatic. The father has previously received 2 doses of COVID‐19 vaccine, while the mother has not been vaccinated. The patient received the first doses of IV ampicillin 50 mg/kg/dose and cefotaxime 50 mg/kg/dose in the emergency department, along with IV fluid and paracetamol for fever. Next, she was admitted to the pediatric ward of Hamad general hospital, a tertiary hospital and one of the main teaching hospitals in Qatar. Shortly after that, she was noticed to have frothy bloody secretion coming out of her mouth; then suddenly, she developed cardiopulmonary arrest. CPR was initiated, and the patient was intubated. She was found to have a pulmonary hemorrhage, as evidenced by the fresh blood from the endotracheal tube and the X‐ray findings of ground‐glass opacities and dense consolidation (Figure 1). Suction yielded approximately 30 ml of bloody secretion. She was given adrenaline and cold normal saline to control the bleeding and transferred to PICU for further care. After initial brief stabilization, the patient started deteriorating, requiring escalation of respiratory support to HFOV. Her sensorium had improved, necessitating initiation of IV continuous sedation with midazolam infusion, as the child was requiring high ventilation pressures and oxygen requirements. One dose of 0.3 mg IV cisatracurium was given; however, the patient did not require a continuous infusion of any paralytic agent. Given that the patient had a pulmonary hemorrhage and severe coagulopathy, ECMO was not initiated. The patient continued to deteriorate and developed bilateral pneumothorax requiring bilateral chest tube insertion (Figure 2). After chest tube insertion, there was a mild improvement in oxygenation with a reduction of FiO_2_ to 0.8 transiently, but it was again increased back to 1.0 due to desaturation. The patient was on the maximum ventilatory settings of MAP of 28, frequency of 8.0, and amplitude of 47, but she kept having frequent desaturation, requiring frequent manual bag to tube ventilation. Echocardiography was done and showed good cardiac function, with no evidence of congenital heart disease, including patent ductus arteriosus, septal defects, and valvular abnormalities. Later, she started developing progressive hypotension, that required support with maximum doses of inotropes. Adrenaline doses were increased from 0.05 μg/kg/min up to 1.8 μg/kg/min. In addition, noradrenaline was started at 0.1 μg/kg/min and increased to 0.5 μg/kg/min. Her urine output started to decrease, for which IV furosemide bolus followed by continuous infusion were started with no response. Blood investigations showed a severe DIC picture. She received platelet transfusion, packed RBC transfusion, fresh frozen plasma, and was empirically covered with meropenem and vancomycin along with remdesivir and dexamethasone for COVID‐19 pneumonia.

**TABLE 1:** Laboratory investigations

Laboratory test	Result	Reference range
*Laboratory test on admission*		
CBC		
WBC	8.7 10^3^/μl	(6.0–16.0) 10^3^/μl
Hgb	12.5 g/dl	(11.1–14.1) g/dl
HCT	35.4%	(30.0–38.0)%
MCV	90.5 fl	(72.0–84.0) fl
MCH	32 pg	(25.0–29.0) pg
Platelet	397 10^3^/μl	(200–550) 10^3^/μl
CSF		
CSF WBC	6/μl	0–5/μL
CSF RBC	3000/μl	0–5/μl
CSF glucose	2.1 mmol/L	(3.33–4.44) mmol/L
CSF protein	0.8 g/L	(0.15–0.45) g/L
CSF viral PCR	Negative	
CSF Culture	No growth	
Other		
Ammonia	79 μmol/L	0–80 μmol/L
Blood culture	No growth for 1 day	
Urine culture	No growth	
Blood virology PCR panel	Negative	
*Laboratory tests after 9 h of admission*		
Ferritin	38,530 μg/L	0–45,038,530 μg/L
IL‐6	1697 pg/L	‐
D‐dimer	>35 mg/L	0–0.46 mg/L
Fibrinogen	<3 g/L	1.6–4.6 g/L
INR	4	—
Urea	6.2 mmol/L	0.8–5.5 mmol/L
Cr	76 μmol/L	—
*Laboratory tests after 12 h of admission*		
D‐dimer	>180 mg/L	0–0.46 mg/L
Fibrinogen	<0.3 g/L	1.6–4.6 g/L
INR	3.8	—
Platelets	79 10^3^/μl	170–500 10^3^/μl
Lactate	13 mmol/L	0–3 mmol/L

Eventually, the child developed progressive desaturation, hypotension, and poor perfusion. Blood gases showed worsening metabolic acidosis. She eventually developed cardiac arrest and was declared dead.

## 3 DISCUSSION

Severe acute respiratory syndrome coronavirus 2 (SARS‐CoV‐2) is the third virus of the twenty‐first century to become a global concern.^2^ Qatar, a country that embraces a population of variable nationalities and ethnicities, was hit by the third wave of COVID‐19 infection in early January 2022, the time at which the patient and her family in this case report acquired COVID‐19 infection. Though the patient was not tested for the exact variant of COVID‐19 virus, we know that 95% of the tested cases at that period were of Omicron variant. More specifically, of the tested omicron infections with confirmed subvariant status, 23.8% were BA.1 and 76.2% were BA.2.^12^

The clinical picture of COVID‐19 infection in the pediatric population seems more indistinct and less severe than in adults, with the most common symptoms being fever, cough, dyspnea, and malaise.^3^ These symptoms are extremely common among children with a variety of respiratory diseases, which they are highly susceptible to due to their developing immune systems. This is thought to be a contributing factor to the delayed presence of published pediatric cases with COVID‐19 infection and their particular ways of disease presentation.^4^ Children are susceptible to infection with COVID‐19 virus as it is mainly transmitted via respiratory droplets.^5^ The incubation period in children is similar to adults and is about 1–14 days, up to 24 days. Children, generally, have immature immunity, and some exhibit a long incubation period after SARS‐CoV‐2 infection.^6^

Newborns can also be infected with SARS‐CoV‐2 due to the immaturity of their immune systems; however, uncommon presentations have been associated with this age group.^2^ In the few published cases of COVID‐19 in neonates, the presentation was that of late neonatal sepsis; interestingly, the lung involvement was not as common as in the older children and adults.^7^

Our patient presented in her neonatal period with pulmonary hemorrhage, which has been reported in adults, but rarely in children with COVID‐19 infection.^8^ It is such a diagnostic challenge because it can be caused by multiple alternative diagnoses, such as chest infections and ANCA vasculitis. However, history, examination, and workup can give a clue to whether the pulmonary hemorrhage is caused by diseases other than COVID‐19 infection. For example, a key distinguishing feature between ANCA‐positive vasculitis and COVID‐19 infection in adults and children who can cough is the presence of hemoptysis. The literature suggests that hemoptysis is uncommon in COVID‐19 and has a symptom prevalence of 2%.^9^

Regarding the etiology of pulmonary hemorrhage in patients with COVID‐19, some case reports in adults suggested that patients with COVID‐19 infection had increased inflammatory states that led to developing vasculitis and consequently pulmonary hemorrhage.^10^ Autopsies performed on deceased adult patients revealed that pulmonary hemorrhage was interestingly associated with the inappropriate formation of thrombi. Furthermore, they showed the signs of diffuse alveolar damage with a rich infiltrate of inflammatory cells, which could contribute to damage to small alveolar vessels.^1^ Due to the rarity of cases of pulmonary hemorrhage in pediatrics, data about presumed etiology are limited and mostly adopted from adults. COVID‐19 virus keeps showing itself in many unfamiliar ways, which leaves physicians in a challenging situation. The paucity of the cases often makes extensive investigations hard to achieve. However, it is necessary to rule out causes such as other viral infections, bacterial diseases, and states of coagulopathy before we can assume hemorrhage is caused primarily by the COVID‐19 virus. The patient in our case underwent screening for infections and coagulation disorder and came out negative. Her young age and acute presentation make rheumatological diseases extremely unlikely. To the best of our knowledge, this is the youngest age at which a patient with COVID‐19 infection developed pulmonary hemorrhage with no other underlying cause of it.

Early bronchoalveolar lavage (BAL) is the diagnostic test needed to confirm diffuse alveolar hemorrhage. The gold standard is the sequential rise in red blood cells or hemosiderin‐laden macrophages on repeat BAL.^9^ This invasive procedure was not done in our case for two main reasons. First, she was clinically unstable to perform bronchoscopy. Second, the procedure is highly aerosol‐generating that should only be performed in the most necessary of cases, to minimize the potential risk of COVID‐19 transmission.^11^

Finally, it became clear that COVID‐19 infection has the potential to cause severe and unusual complications, particularly in the youngest age groups. This should alert physicians to closely monitor their COVID‐19 positive neonates presenting with certain conditions, such as neonatal sepsis and respiratory illnesses. In addition, there should be a lower threshold for admission to PICU at any signs of deterioration. Management of these cases should be preferably done in specialized tertiary centers, where they can be placed in negative pressure rooms, and provided with the highest available level of care. Multidisciplinary team approach is needed to manage neonates with COVID‐19‐induced pulmonary hemorrhage. That includes a pulmonologist with expertise in bronchoscopy in critical situations, an intensive cardiologist, an infectious disease specialist, PICU intensivists, respiratory therapists, and clinical pharmacists.

## 4 CONCLUSION

Pulmonary hemorrhage has been reported in adults but rarely in children. Some reports in adults suggested that patients with COVID‐19 infection had an increased inflammatory state that led to the development of vasculitis and pulmonary hemorrhage. While many of the cases of COVID‐19 infection in children are mild, fatal complications such as pulmonary hemorrhage can be present, adding new challenges to the management of this novel and widely spreading virus.

## AUTHOR CONTRIBUTIONS

AA, KE, TM, WA, HA, AS, MA, and AJ collected the data, performed literature search, and prepared the manuscript (draft and final editing). All authors read and approved the final manuscript.

## CONFLICT OF INTEREST

The authors declare that they have no competing interests.

## ETHICAL APPROVAL

This case report was approved by the Medical Research Center at Hamad Medical Corporation (MRC‐04‐22‐053) with a waiver of ethical approval.

## CONSENT

The consent for publication was obtained from the patient's guardian.

## Data Availability

All data generated or analyzed during this study are included in this published article.
